# OMICS-based personalized oncology: if it is worth doing, it is worth doing well!

**DOI:** 10.1186/1741-7015-11-221

**Published:** 2013-10-17

**Authors:** Daniel F Hayes

**Affiliations:** 1 Breast Oncology Program, University of Michigan Comprehensive Cancer Center, 6312 Cancer Center, 1500 E. Medical Center Drive, Ann Arbor, MI 48109-0942, USA

**Keywords:** Tumor biomarker tests, Clinical investigation

## Abstract

The era of Personalized Medicine implies getting the right treatment to the right patient at the right schedule and dose at the right time. Tumor biomarker tests are keys to accomplishing this goal successfully. However, much of the translational research regarding tumor biomarker tests has been haphazard, often using data and specimen sets of convenience and ignoring many of the principles of the scientific method. In papers published simultaneously in *BMC Medicine* and *Nature*, McShane and colleagues have proposed a checklist of criteria that should be followed by investigators planning to conduct prospective clinical trials directed towards generating high levels of evidence to demonstrate whether a tumor biomarker test has clinical utility for a specific context. These criteria were generated in response to a roadmap reported by a committee convened by the U.S. Institute of Medicine for generation of omics-based biomarker tests. Taken together with several other initiatives to increase the rigor of tumor biomarker research, these criteria will increase the perception of value for tumor biomarker test research and application in the clinic.

Please see related article: http://www.biomedcentral.com/1741-7015/11/220.

## Background

Over the last few years, one cannot open a clinical journal without an article ostensibly addressing some component of 'Personalized' or 'Individualized' or 'Precision' medicine. This trend is particularly evident in the field of oncology. Personalized oncology is simply defined as 'getting the right treatment to the right patient at the right dose and schedule at the right time [1]. These papers usually report some component of the use of tumor biomarkers to better select which patients might be more likely to benefit from a given clinical care strategy, by virtue of either being more likely to respond or less likely to suffer toxicities.

The use of diagnostics to better treat patients is as old as medicine itself. Within the field of oncology, pathologic findings have been used to direct various chemotherapy regimens based on tissue of origin. More precisely, estrogen receptor (ER) has served as a predictive biomarker in breast cancer for selection of endocrine therapy since the mid-1970s [2,3]. In the last 15 years HER2 has joined ER in breast cancer as a predictive factor, in this case serving as a marker for therapies directed towards the HER2 protein [4,5]. More recently, other examples of useful biomarkers to direct novel targeted treatments in colorectal, lung, and hematologic malignancies have been reported.

Perhaps the most compelling stimulus for the interest in personalized medicine has grown from the omics-revolution of the last 15 years [6]. Based on the cloning of the human genome in the latter part of the 1990s, fascinating technologies that allow the simultaneous measurement of thousands of analytes (RNA, proteins, metabolites and so on) have been coupled with sophisticated bioinformatics to permit development of multi-parameter signatures that correlate with either biological or clinical phenotypes and outcomes.

Sadly, in spite of these amazing advances, only a few diagnostics have been adopted successfully into routine clinical care of patients with cancer [7]. Many reasons for the disappointing output of clinically useful tumor biomarkers have been addressed over the last decade [6,8-12]. This commentary summarizes the reasons for the low output of clinically useful tumor biomarkers and also discusses the ways in which this is being addressed.

### Addressing the lack of clinically useful tumor biomarkers

In order that tumor biomarkers have clinical impact, there are two issues that must be addressed. First, it is important to distinguish a tumor biomarker from a tumor biomarker test. The term 'biomarker' usually refers to a biological factor or process that is identified in malignant but not normal tissues or other biospecimens. In contrast, a tumor biomarker *test* is a specific assay for the biomarker. Indeed, it is possible that many tests may be developed for one tumor biomarker. HER2 offers an ideal example. HER2 can be over-expressed at the message and/or protein level, which can be a result of either amplification or regulatory control [4,5]. Furthermore, activating HER2 mutations have recently been reported in breast cancers that have normal copy gene numbers [13]. Circulating extra-cellular domain of the HER2 protein levels may be detected in serum [7]. There are many tests that have been generated to identify and quantify these different circumstances, each with variable use in the clinic. Regardless, it is essential that any tumor biomarker that is to be used to direct care is accurate, reproducible, and reliable - put simply, it must have analytical validity [10].

Second, a tumor biomarker test must have clinical utility - a term coined by the Evaluation of Genomic Applications in Practice and Prevention (EGAPP) Initiative in 2009 [10]. Clinical utility does not just imply that the tumor biomarker test divides a population into two separate groups with statistical significance (a circumstance EGAPP termed 'clinical validity'). Rather, clinical utility is achieved if high levels of evidence have been generated that consistently demonstrate that applying the tumor biomarker test results in improved outcomes for the patient when compared to not using the assay to direct care. High levels of evidence may come either from prospectively directed clinical trials [14,15] or from 'prospective retrospective' studies using archived specimens derived from previously conducted clinical trials [11].

**Figure 1:**
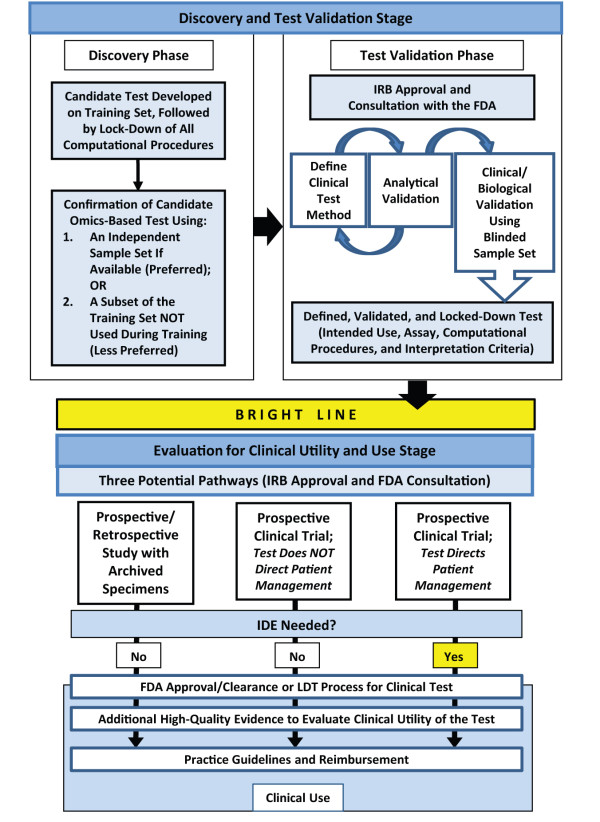
**Institute of Medicine Roadmap for omics-based tumor biomarker test development.**The first stage encompasses discovery of a biologically, and perhaps clinically, interesting omics-based tumor biomarker and development of an analytically-validated tumor biomarker test with clinical validity. The second stage is directed towards evaluation for clinical utility and use of the tumor biomarker test, either in a prospective-retrospective study using archived specimens or in prospective clinical trials designed to 'test the test' for its intended clinical use. Reprinted with permission from reference [6] by the National Academy of Sciences, Courtesy of the National Academies Press, Washington, D.C.

Although these concepts apply to any diagnostic in general, and to any tumor biomarker specifically, the advent of omics-based tumor biomarker studies has led to a plethora of reports of putative assays that are highly confounded by the astounding number of data points applied to a vanishingly small patient dataset. Recently, in a highly publicized situation, an unstable set of signatures that had no evidence of analytical stability was used to direct specific therapies within prospective clinical trials. This unfortunate set of circumstances led to a comprehensive review by a committee of experts, convened by the United States Institute of Medicine (IOM), of the translation of omics-based tests to clinical trials, and ultimately to clinical care [6]. The IOM committee generated a roadmap for investigators to follow during development of a putative new omics-based tumor biomarker test. This roadmap leads investigators through three separate but linked stages of development: Discovery, Test Development, and Evaluation of Clinical Utility and Use (Figure1).

### A roadmap for the improvement of tumor biomarker research

In a correspondence article in *BMC Medicine*[16], and in an accompanying companion paper published simultaneously in *Nature*[17], McShane and her colleagues extend these efforts to improve tumor biomarker research by providing a checklist of criteria for the use of omics-based predictors in clinical trials. This checklist grew out of a workshop convened by the United States National Cancer Institute (NCI), specifically to consider the recommendations from the IOM committee. The criteria pertain to all three stages of the IOM roadmap (Figure1), but they are especially pertinent to investigators who have elected to cross the 'Bright Line' illustrated in Figure1 that distinguishes the Discovery and Test Validation Stage from the Evaluation of Clinical Utility and Use Stage. The criteria are particularly directed towards those investigators who have chosen the strategy of conducting a prospective clinical trial to 'test the test' (see Figure1, middle and far right side of bottom panel). Of note, many of these criteria are not novel - indeed, McShane has worked tirelessly with many colleagues calling for transparent reporting of pre-analytical (the 'BRISQ criteria') and study design and analytical (the 'REMARK criteria') issues in papers describing tumor biomarker results [8,9,12]. However, the current publication is a comprehensive, yet concise, set of criteria about which any investigator considering a clinical trial to generate high levels of evidence for clinical utility of a tumor biomarker test must be aware.

Why is this checklist needed? Because, 'A Bad Tumor Marker Is as Bad as a Bad Drug [18]. Ironically, over the last century, the scientific community has developed very strict criteria for scientific discovery and reporting of both basic laboratory research and clinical trial investigations. For example, basic laboratory researchers follow a strict code of scientific methods ensuring proper experimental design, using appropriate control conditions and insisting on reproducibility. Likewise, clinical therapeutic trialists use prospectively written protocols that describe the objectives and subject eligibility for the trial and stipulate how the therapeutic agent will be prepared and administered. Moreover, the protocol states in great detail what endpoints will be evaluated and how they will be measured (with specific scales and criteria) and, finally, provides a specific statistical plan that must be followed during and at the conclusion of the trial. These features are designed to minimize unintentional, or intentional, biases and reporting, in an effort to produce believable, reproducible results - the hallmark of the scientific method.

For a variety of reasons, investigators who translate putative biomarkers from basic research to clinical studies have often ignored some of these fundamental principles of the scientific process. Rather, tumor biomarker investigations have, too often, been studies of convenience, in which the investigators have applied an assay (which may or may not have analytical validity) to some available patient specimens, observed separation in some outcome of the population at hand with a *P* value <0.05, and declared victory. Although such a study may suggest clinical validity, the results rarely, if ever, demonstrate clinical utility. Unfortunately, very few investigators take the next step across the Bright Line into the Evaluation for Clinical Utility and Use Stage outlined in the IOM roadmap (Figure1). Consequently, while thousands of manuscripts have been published in the peer-reviewed literature, few tumor biomarker tests have sufficiently high levels of evidence of clinical utility to warrant recommendations for use to direct patient care. The NCI Workshop criteria proposed by McShane *et al*, which are carefully explained in the *BMC Medicine* Explanation and Elaboration article [16], represent a further effort to provide a 'tour guide' to accompany the roadmap laid out by the IOM Committee.

## Conclusions

Clearly, the scientific, regulatory, reimbursement, medical and lay communities do not value tumor biomarker tests to the same extent as cancer therapeutics. Recently, many of these concepts have been distilled into what has been designated a 'vicious cycle' that emerges from the devaluation of tumor biomarker research and clinical application. Several transformative recommendations were suggested to break the cycle and create a 'virtuous cycle [19]. If we do not approach this problem systematically, as suggested by the IOM and the NCI Working Committee, the promise of personalized oncology will never materialize. Worse, assays of questionable value will be marketed to the public, *caveat emptor*, possibly resulting in unknown amounts of over- and under-treatment. It is essential that investigators considering generation of studies to develop Level 1 evidence supporting clinical utility of tumor biomarker tests be aware, cite and, more importantly, adhere to the criteria put forward by the NCI Workshop Committee.

## Competing interests

Dr. Hayes has received research support from Veridex/Janssen (subsidiaries of Johnson and Johnson) to conduct laboratory and clinical studies of circulating tumor cells. Dr. Hayes has three patents pending regarding clinical use of circulating tumor cells. He serves on the advisory boards of Oncimmune LLC and Inbiomotion, LLC, and has stock options in both of these companies, which are both manufacturers of potential tumor biomarker tests.

## Authors' information

Dr. Daniel F. Hayes is the Stuart B. Padnos Professor of Breast Cancer Research and Clinical Director of the Breast Oncology Program at the University of Michigan Comprehensive Cancer Center and has been a leader in tumor biomarker development, evaluation, and clinical utility, including assays for circulating proteins and tumor cells, tissue-based and germ line factors associated with drug metabolism and activity. He is Chair or Co-Chair of the Breast Cancer Translational Medicine Committee of the Southwest Oncology Group, the Correlative Science Committee of the North American Breast Cancer Group (formerly known as the Intergroup), the TransOx Correlative Science Committee of the Early Breast Cancer Trialists' Collaborative Group, the Consortium on Breast Cancer Pharmacogenomics (COBRA), and the American Society of Clinical Oncology (ASCO) Tumor Marker Guidelines Committee. He was a member of the Committee on the Review of Omics-Based Tests for Predicting Patient Outcomes in Clinical Trials, convened by the Institute of Medicine of the National Academies.

## References

[B1] Schilsky RL Personalizing cancer care: American Society of Clinical Oncology presidential address 2009 J Clin Oncol 2009 27 3725 3730 10.1200/JCO.2009.24.6827 19581526

[B2] Hammond ME Hayes DF Dowsett M Allred DC Hagerty KL Badve S Fitzgibbons PL Francis G Goldstein NS Hayes M Hicks DG Lester S Love R Mangu PB McShane L Miller K Osborne CK Paik S Perlmutter J Rhodes A Sasano H Schwartz JN Sweep FC Taube S Torlakovic EE Valenstein P Viale G Visscher D Wheeler T Williams RB American Society of Clinical Oncology/College of American Pathologists guideline recommendations for immunohistochemical testing of estrogen and progesterone receptors in breast cancer (unabridged version) Arch Pathol Lab Med 2010 134 e48 72 20586616 10.5858/134.7.e48

[B3] Hammond ME Hayes DF Dowsett M Allred DC Hagerty KL Badve S Fitzgibbons PL Francis G Goldstein NS Hayes M Hicks DG Lester S Love R Mangu PB McShane L Miller K Osborne CK Paik S Perlmutter J Rhodes A Sasano H Schwartz JN Sweep FC Taube S Torlakovic EE Valenstein P Viale G Visscher D Wheeler T Williams RB **American Society of Clinical Oncology/College Of American Pathologists guideline recommendations for immunohistochemical testing of estrogen and progesterone receptors in breast cancer** J Clin Oncol 2010 28 2784 2795 10.1200/JCO.2009.25.6529 20404251 PMC2881855

[B4] Wolff AC Hammond ME Schwartz JN Hagerty KL Allred DC Cote RJ Dowsett M Fitzgibbons PL Hanna WM Langer A McShane LM Paik S Pegram MD Perez EA Press MF Rhodes A Sturgeon C Taube SE Tubbs R Vance GH van de Vijver M Wheeler TM Hayes DF American Society of Clinical Oncology; College of American Pathologists American Society of Clinical Oncology/College of American Pathologists guideline recommendations for human epidermal growth factor receptor 2 testing in breast cancer J Clin Oncol 2007 25 118 145 17159189 10.1200/JCO.2006.09.2775

[B5] Wolff AC Hammond ME Schwartz JN Hagerty KL Allred DC Cote RJ Dowsett M Fitzgibbons PL Hanna WM Langer A McShane LM Paik S Pegram MD Perez EA Press MF Rhodes A Sturgeon C Taube SE Tubbs R Vance GH van de Vijver M Wheeler TM Hayes DF American Society of Clinical Oncology/College of American Pathologists American Society of Clinical Oncology/College of American Pathologists guideline recommendations for human epidermal growth factor receptor 2 testing in breast cancer Arch Pathol Lab Med 2007 131 18 43 19548375 10.5858/2007-131-18-ASOCCO

[B6] Institute of Medicine Evolution of Translational omics: Lessons Learned and the Path Forward 2012 Washington, DC: The National Academies Press 24872966

[B7] Harris L Fritsche H Mennel R Norton L Ravdin P Taube S Somerfield MR Hayes DF Bast RC Jr American Society of Clinical Oncology 2007 update of recommendations for the use of tumor markers in breast cancer J Clin Oncol 2007 25 5287 5312 10.1200/JCO.2007.14.2364 17954709

[B8] McShane LM Altman DG Sauerbrei W Taube SE Gion M Clark GM Reporting recommendations for tumor marker prognostic studies J Clin Oncol 2005 23 9067 9072 10.1200/JCO.2004.01.0454 16172462

[B9] Moore HM Kelly A McShane LM Vaught J Biospecimen reporting for improved study quality (BRISQ) Clin Chim Acta 2012 413 1305 10.1016/j.cca.2012.04.013 22543057

[B10] Teutsch SM Bradley LA Palomaki GE Haddow JE Piper M Calonge N Dotson WD Douglas MP Berg AO The Evaluation of Genomic Applications in Practice and Prevention (EGAPP) Initiative: methods of the EGAPP Working Group Genet Med 2009 11 3 14 10.1097/GIM.0b013e318184137c 18813139 PMC2743609

[B11] Simon RM Paik S Hayes DF Use of archived specimens in evaluation of prognostic and predictive biomarkers J Natl Cancer Inst 2009 101 1446 1452 10.1093/jnci/djp335 19815849 PMC2782246

[B12] McShane L Hayes DF Publication of tumor marker research results: the necessity for complete and transparent reporting J Clin Oncol 2012 30 4223 4232 10.1200/JCO.2012.42.6858 23071235 PMC3504327

[B13] Bose R Kavuri SM Searleman AC Shen W Shen D Koboldt DC Monsey J Goel N Aronson AB Li S Ma CX Ding L Mardis ER Ellis MJ Activating HER2 mutations in HER2 gene amplification negative breast cancer Cancer Discov 2013 3 224 237 10.1158/2159-8290.CD-12-0349 23220880 PMC3570596

[B14] Sargent DJ Conley BA Allegra C Collette L Clinical trial designs for predictive marker validation in cancer treatment trials J Clin Oncol 2005 23 2020 2027 10.1200/JCO.2005.01.112 15774793

[B15] Freidlin B McShane LM Polley MY Korn EL Randomized phase II trial designs with biomarkers J Clin Oncol 2012 30 3304 3309 10.1200/JCO.2012.43.3946 22869885 PMC3434989

[B16] McShane LM Cavenagh MM Lively T Eberhard DA Bigbee WL Williams MP Mesirov JP Polley MY Kim KY Tricoli JV Criteria for the use of omics-based predictors in clinical trials: explanation and elaboration BMC Med 2013 11 220 24228635 10.1186/1741-7015-11-220 PMC3852338

[B17] McShane LM Cavenagh MM Lively T Eberhard DA Bigbee WL Williams MP Mesirov JP Polley MY Kim KY Tricoli JV Criteria for the use of omics-based predictors in clinical trials Nature 2013 502 317 320 10.1038/nature12564 24132288 PMC4180668

[B18] Hayes DF Genome to bedside: Lost in translation - the importance of demonstrating analytical validity and high levels of evidence of clinical utility before adopting a tumor biomarker into routine clinical use Breastin press

[B19] Hayes DF Allen J Compton C Gustavsen G Leonard DG McCormack R Newcomer L Pothier K Ransohoff D Schilsky RL Sigal E Taube SE Tunis SR Breaking a vicious cycle Sci Transl Med 2013 5 196cm196 10.1126/scitranslmed.3005950 23903752

